# Supplementary figures and images for: The reproductive inhibitory effects of levonorgestrel, quinestrol, and EP-1 in Brandt’s vole (Lasiopodomys brandtii)
Source: PeerJ. 2020 Jun 11;8:e9140. doi: 10.7717/peerj.9140 (PMC7293854; doi:10.7717/peerj.9140)

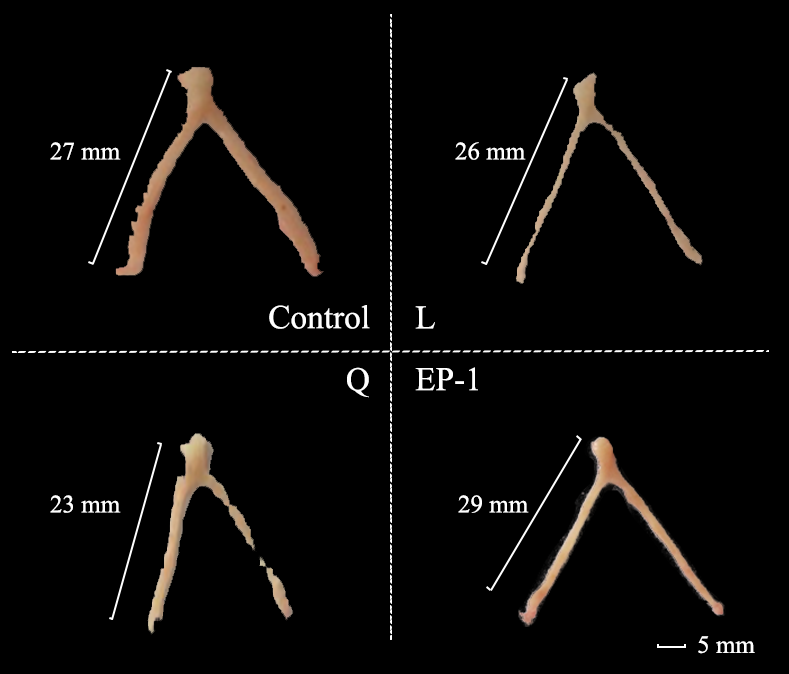

Supplement: Supplemental Information 1 [file peerj-08-9140-s001.png]

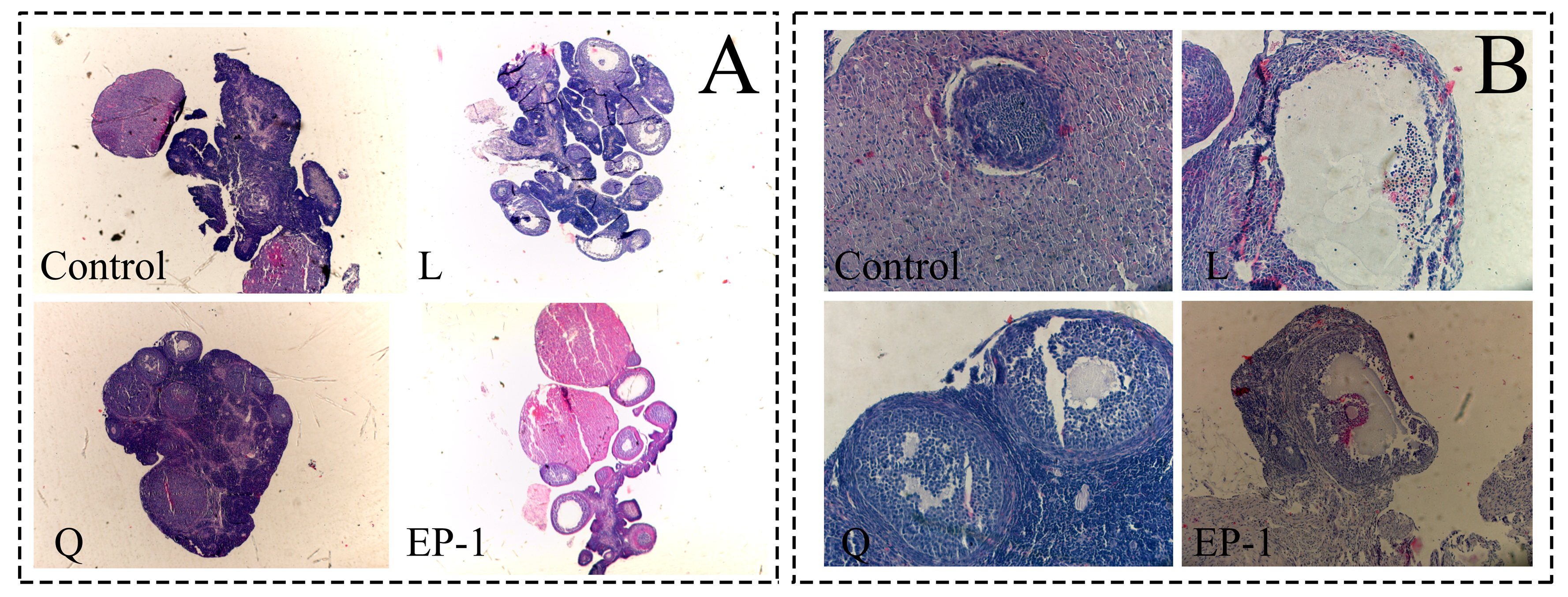

Supplement: Supplemental Information 2 [file peerj-08-9140-s002.png]

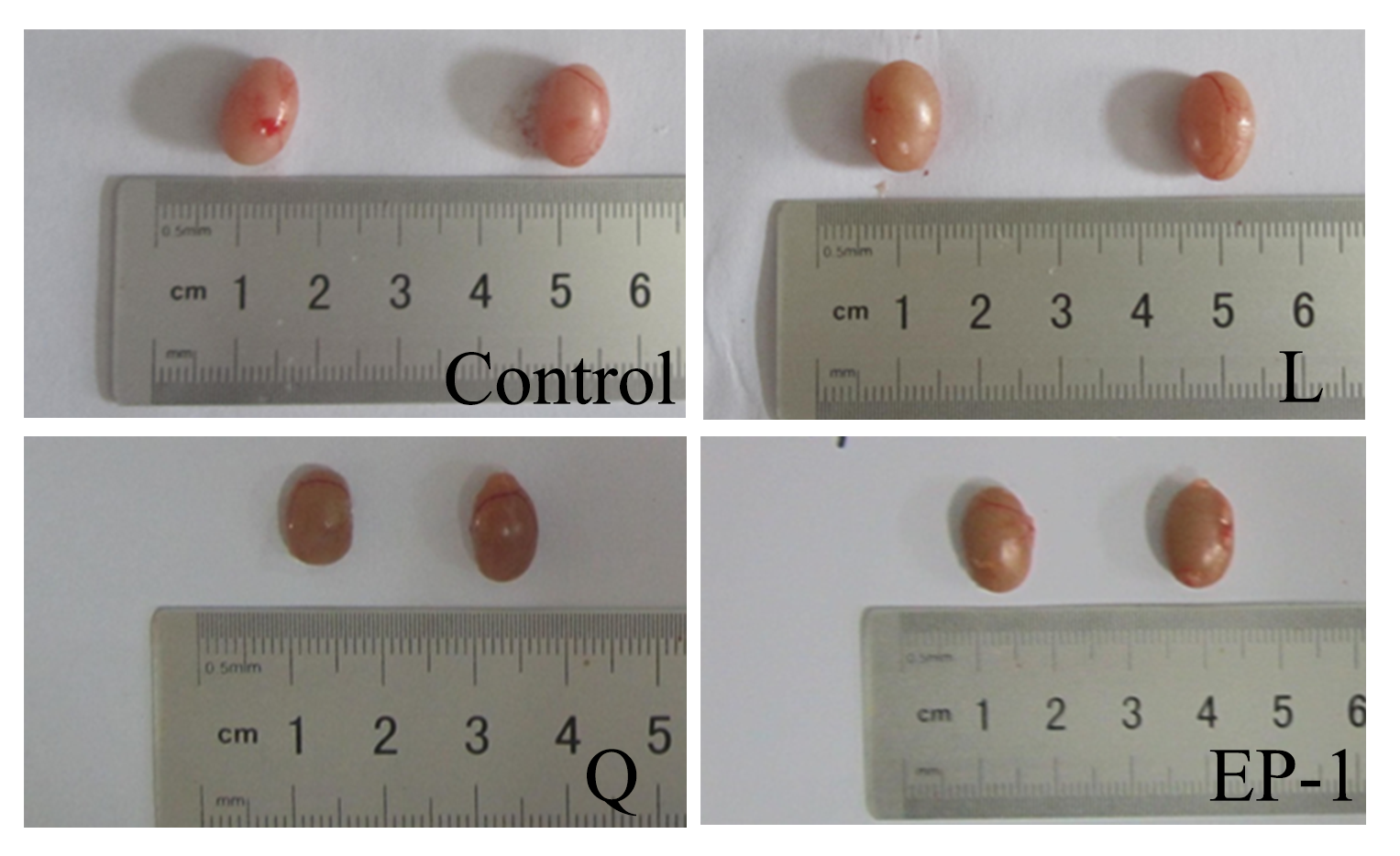

Supplement: Supplemental Information 3 [file peerj-08-9140-s003.png]

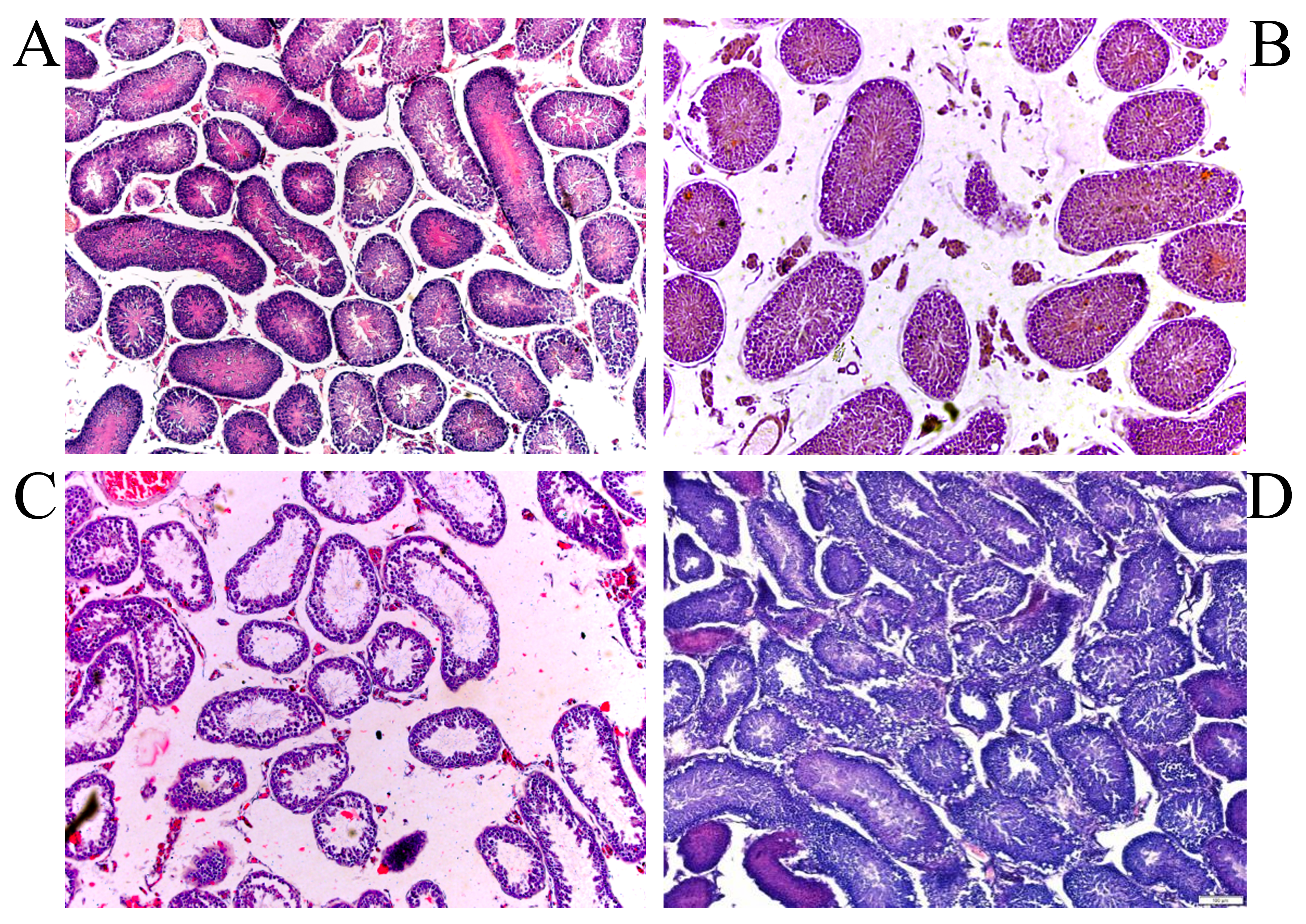

Supplement: Supplemental Information 4 [file peerj-08-9140-s004.png]
