# Supplementary material for: The reproductive inhibitory effects of levonorgestrel, quinestrol, and EP-1 in Brandt’s vole (Lasiopodomys brandtii)
Source: PeerJ. 2020 Jun 11;8:e9140. doi: 10.7717/peerj.9140 (PMC7293854; doi:10.7717/peerj.9140)
Supplement: Supplemental Information 5 [file peerj-08-9140-s005.docx]

**Table S1**. Body mass changes of voles in the three fertility control agents treated groups and control group.

| **Group** | **No.** | **Gender** | **0 day (g)** | **7 day (g)** | **14 day (g)** | **21 day (g)** | **28 day (g)** |
| --- | --- | --- | --- | --- | --- | --- | --- |
| **L group** | 1 | Femal | 45.0 | 46.6 | 47.3 | 50.6 | 49.4 |
|  | 2 | Femal | 50.8 | 52.7 | 53.0 | 56.8 | 55.5 |
|  | 3 | Femal | 45.8 | 47.8 | 48.1 | 51.8 | 50.7 |
|  | 4 | Femal | 46.0 | 48.0 | 48.5 | 52.0 | 51.2 |
|  | 5 | Femal | 50.2 | 52.2 | 52.8 | 56.5 | 55.4 |
|  | 6 | Femal | 52.3 | 54.6 | 55.4 | 59.5 | 58.5 |
|  | 7 | Femal | 51.8 | 54.1 | 54.5 | 58.4 | 57.3 |
|  | 8 | Male | 45.4 | 47.2 | 47.7 | 51.3 | 50.5 |
|  | 9 | Male | 46.6 | 48.5 | 48.8 | 52.7 | 51.8 |
|  | 10 | Male | 53.0 | 55.3 | 56.1 | 60.4 | 59.3 |
|  | 11 | Male | 44.8 | 46.8 | 47.4 | 51.1 | 50.2 |
|  | 12 | Male | 53.4 | 55.4 | 56.0 | 60.0 | 59.0 |
|  | 13 | Male | 48.2 | 50.1 | 50.8 | 54.5 | 53.3 |
|  | 14 | Male | 43.6 | 45.4 | 45.9 | 49.1 | 48.1 |
| **Q Group** | 1 | Femal | 48.1 | 41.9 | 39.5 | 42.5 | 44.1 |
|  | 2 | Femal | 47.8 | 41.7 | 39.5 | 42.6 | 44.3 |
|  | 3 | Femal | 45.1 | 39.6 | 37.3 | 40.0 | 41.4 |
|  | 4 | Femal | 51.2 | 44.7 | 42.1 | 45.2 | 46.7 |
|  | 5 | Femal | 41.4 | 36.1 | 34.0 | 36.3 | 37.5 |
|  | 6 | Femal | 49.6 | 43.4 | 40.9 | 44.1 | 45.6 |
|  | 7 | Femal | 40.1 | 35.0 | 32.9 | 35.1 | 36.3 |
|  | 8 | Male | 53.4 | 46.8 | 44.4 | 47.5 | 49.3 |
|  | 9 | Male | 40.2 | 35.0 | 32.7 | 35.2 | 36.4 |
|  | 10 | Male | 53.5 | 46.9 | 44.4 | 47.5 | 49.1 |
|  | 11 | Male | 42.0 | 36.5 | 34.4 | 36.9 | 37.9 |
|  | 12 | Male | 45.2 | 39.7 | 37.3 | 39.9 | 41.0 |
|  | 13 | Male | 46.5 | 40.4 | 38.1 | 40.9 | 42.1 |
|  | 14 | Male | 43.1 | 37.8 | 35.9 | 38.7 | 39.9 |
| **EP-1** | 1 | Femal | 50.1 | 47.3 | 45.4 | 45.6 | 46.8 |
|  | 2 | Femal | 45.7 | 43.1 | 41.7 | 41.8 | 43.0 |
|  | 3 | Femal | 45.4 | 42.9 | 41.5 | 41.7 | 42.9 |
|  | 4 | Femal | 48.7 | 46.2 | 44.6 | 45.0 | 46.1 |
|  | 5 | Femal | 48.1 | 45.7 | 44.0 | 44.1 | 45.2 |
|  | 6 | Femal | 49.0 | 46.1 | 44.5 | 44.5 | 45.4 |
|  | 7 | Femal | 53.4 | 50.4 | 48.8 | 48.9 | 49.9 |
|  | 8 | Male | 52.2 | 49.3 | 47.3 | 47.8 | 48.8 |
|  | 9 | Male | 48.2 | 45.5 | 43.9 | 44.1 | 45.2 |
|  | 10 | Male | 53.5 | 50.8 | 49.0 | 49.3 | 50.5 |
|  | 11 | Male | 51.0 | 48.0 | 46.6 | 46.6 | 47.6 |
|  | 12 | Male | 54.8 | 52.0 | 50.2 | 50.4 | 51.8 |
|  | 13 | Male | 50.5 | 47.8 | 46.1 | 46.5 | 47.6 |
|  | 14 | Male | 40.3 | 38.1 | 36.9 | 37.1 | 37.7 |
| **Control** | 1 | Femal | 43.3 | 42.8 | 43.0 | 44.1 | 44.1 |
|  | 2 | Femal | 49.9 | 49.3 | 49.4 | 50.8 | 50.6 |
|  | 3 | Femal | 53.6 | 52.8 | 52.9 | 54.4 | 54.1 |
|  | 4 | Femal | 40.6 | 40.1 | 40.2 | 41.2 | 41.4 |
|  | 5 | Femal | 48.9 | 48.0 | 48.1 | 49.1 | 49.0 |
|  | 6 | Femal | 41.3 | 40.4 | 40.4 | 41.6 | 41.6 |
|  | 7 | Femal | 40.0 | 39.4 | 39.8 | 40.8 | 40.6 |
|  | 8 | Male | 42.3 | 41.5 | 41.9 | 42.7 | 42.9 |
|  | 9 | Male | 51.7 | 50.8 | 51.0 | 52.5 | 52.4 |
|  | 10 | Male | 47.1 | 46.3 | 46.6 | 47.5 | 47.7 |
|  | 11 | Male | 44.8 | 44.0 | 44.0 | 45.3 | 45.2 |
|  | 12 | Male | 42.0 | 41.2 | 41.4 | 42.4 | 42.5 |
|  | 13 | Male | 54.4 | 53.5 | 53.7 | 55.1 | 55.2 |
|  | 14 | Male | 44.2 | 43.7 | 44.0 | 45.0 | 45.3 |
